# NRF2 Regulates *PINK1* Expression under Oxidative Stress Conditions

**DOI:** 10.1371/journal.pone.0142438

**Published:** 2015-11-10

**Authors:** Hitoshi Murata, Hitoshi Takamatsu, Sulai Liu, Ken Kataoka, Nam-ho Huh, Masakiyo Sakaguchi

**Affiliations:** 1 Department of Cell Biology, Okayama University Graduate School of Medicine, Dentistry and Pharmaceutical Sciences, 2-5-1 Shikata-cho, Kita-ku, Okayama 700–8558, Japan; 2 Department of Life Science, Faculty of Science, Okayama University of Science, 1–1 Ridai-cho, Kita-ku, Okayama 700–0005, Japan; North Carolina State University, UNITED STATES

## Abstract

Mutations of the *PTEN-induced putative kinase 1* (*PINK1*) gene are a cause of autosomal recessive forms of Parkinson’s disease. Recent studies have revealed that PINK1 is an essential factor for controlling mitochondrial quality, and that it protects cells from oxidative stresses. Although there has been considerable progress in the elucidation of various aspects of PINK1 protein regulation such as activation, stability and degradation, the transcriptional regulation of *PINK1* mRNA under stress conditions remains unclear. In this study, we found that nuclear factor (erythroid-derived 2)-like 2 (NRF2), an antioxidant transcription factor, regulates *PINK1* expression under oxidative stress conditions. Damaged mitochondria arising from stress conditions induced NRF2-dependent transcription of the *PINK1* gene through production of reactive oxygen species (ROS). Either an ROS scavenger or forced expression of KEAP1, a potent inhibitory partner to NRF2, restricted *PINK1* expression induced by activated NRF2. Transcriptionally up-regulated PINK1 diminished oxidative stress-associated cell death. The results indicate that *PINK1* expression is positively regulated by NRF2 and that the NRF2-PINK1 signaling axis is deeply involved in cell survival.

## Introduction

The *PINK1* gene encodes a 581-amino-acid protein that harbors an N-terminal mitochondrial targeting sequence followed by a putative transmembrane region and a serine/threonine kinase domain [1]. Mutations in the *PINK1* gene can cause familial Parkinson’s disease [1, 2]. PINK1 protects cells against various stresses through activation of Akt via mammalian target of rapamycin complex 2 (mTORC2) [3, 4], regulation of Bcl-xL [5, 6] and elimination of damaged mitochondria [7, 8]. When mitochondria are damaged and lose membrane potential, PINK1 accumulates on the outer membrane of depolarized mitochondria. We have shown that PINK1 forms a complex with Sterile alpha and TIR motif-containing 1 (SARM1) and Tumor necrosis factor receptor-associated factor 6 (TRAF6), and this complex is important for the import of PINK1 to the outer membrane and accumulation of PINK1 on depolarized mitochondria [9]. Accumulated PINK1 recruits Parkin, a PD-linked E3 ubiquitin ligase, and Parkin can mediate the autophagic elimination of depolarized mitochondria [10]. Thus, there has been considerable progress in the elucidation of PINK1 protein regulation, while its transcriptional regulation has been barely explored [11–14]. Forkhead box O3a (FOXO3a) transcription factor has been reported to control *PINK1* gene expression [11]. However, we have found that it is not powerful enough to activate the PINK1 promoter in a line of neuronal cells under oxidative stress conditions, and we therefore aimed to elucidate the complex mechanism of *PINK1* gene expression. By searching the PINK1 promoter, we found the presence of antioxidant responsive elements (ARE), which is the binding sequence of NRF2.

NRF2, a basic leucine zipper (bZIP) transcription factor, is a labile protein that is stabilized under an oxidative stress condition and up-regulates cytoprotective genes, such as those for NAD(P)H-quinone oxidoreductase 1 (*NQO1*), glutathione *S*-transferases (*GST*s), glutamate cysteine ligase (*GCL*), and heme oxygenase 1 (*HO-1*) [15–19]. NRF2 deficiency largely abolishes the constitutive and/or inducible expression of defense enzyme genes in response to oxidative stress. Loss of expression of NRF2 significantly enhances the susceptibility of mice to various oxidative stress-related pathologies, including chemical carcinogenesis, hyperoxia, hemolytic anemia and neurodegenerative diseases [20–23]. Under normal or unstressed condition, NRF2 is kept in the cytoplasm and is rapidly degraded by Kelch-like ECH-associated protein 1 (KEAP1) and Cullin 3 through the ubiquitin-proteasome pathway [24]. Under an oxidative stress condition, oxidants and electrophiles oxidize critical cysteine residues in KEAP1 and then NRF2 is released from KEAP1 [25]. The released NRF2 translocates into the nucleus, forms heterodimers with small Maf proteins, binds to ARE, and activates transcription of target genes [15].

PINK1 is necessary for mitochondrial quality control and contributes to mitophagy [7, 8]. To maintain an appropriate level of PINK1 in cells, transcription of *PINK1* mRNA must be activated. Here, we report that reactive oxygen species (ROS) inactivate KEAP1 and release NRF2 from KEAP1 binding. The released NRF2 activates transcriptional up-regulation of *PINK1* mRNA. In this study, we showed that the NRF2-PINK1 signaling axis has a significant role in cell survival.

## Materials and Methods

### Cells, chemicals and antibodies

SH-SY5Y (ECACC, Salisbury, Wiltshire, United Kingdom), HCT-15, HCT116, HEK293, HEK293T and MCF-7 cells were cultured in DMEM/F-12 medium (Invitrogen, Carlsbad, CA, USA) supplemented with 10% fetal bovine serum. To obtain normal human matured neuronal cells in a culture system, ReproNeuro, a neuron progenitor derived from human iPS cells, was purchased from ReproCELL (Yokohama, Japan) and maintained in ReproNeuro maturation medium for 14 days according to the manufacturer’s instructions. Carbonyl cyanide *m*-chlorophenyl hydrazone (CCCP), 6-hydroxydopamine hydrobromide (6-OHDA), cadmium chloride (CdCl_2_), rotenone, *tert*-Butylhydroquinone (tBHQ), sulforaphane and *N*-acetylcysteine (NAC) were purchased from Sigma-Aldrich (St. Louis, MO, USA). Paraquat, caffeine and curcumin were purchased from Wako Chemicals (Osaka, Japan). The antibodies used were as follows: an antibody against PINK1 (rabbit monoclonal, 6946), an antibody against NRF2 (rabbit monoclonal, 12721), horseradish peroxidase (HRP)-conjugated anti-mouse and anti-rabbit secondary antibodies (Cell Signaling Technologies, Danvers, MA, USA), and an antibody against Tubulin (mouse monoclonal, T5168; Sigma-Aldrich).

### Plasmid constructs

Conventional molecular biological techniques were used to generate the following expression constructs: N-terminal Flag-tagged human wild-type NRF2 and NRF2-deletion mutants of two transcription activation domains (Neh4-5; 111–201 amino acids); N-terminal Flag-tagged Forkhead box O3a (FOXO3a); N-terminal HA-tagged KEAP1; C-terminal HA-tagged human wild-type PINK1 and PINK1 catalytically inactive KDD triple mutant (K219A/D362A/D384A) [26]. The human PINK1 promoter covering a region from -3060 to -1 (3060 bp) was amplified from human genomic DNA (Millipore, Billerica, MA, USA) by PCR and ligated into the pGL4.14 luciferase reporter vector, pGL4.14 [luc2/Hygro], (Promega Biosciences, San Luis Obispo, CA, USA). To establish stable cell lines expressing the PINK1-promoter-Luciferase cassette, the construct (pGL4.14 [luc2/Hygro]-PINK1 promoter [3060 bp]) described above was transfected to SH-SY-5Y cells. After selection with hygromycin, optimal clones were obtained. To analyze the significance of the ARE sites (TGA- - - -GC, 9 bp; ARE1, -2632 to -2624 bp; ARE2, -1742 to -1734 bp; ARE3, -660 to -652 bp; ARE4, -235 to -227 bp) in the PINK1 promoter, the sequence was mutated to TAG- - - -CG. All expression constructs were sequenced to ensure that the fusion was in the correct reading frame and there were no additional mutations.

### Cell transfection and luciferase reporter assay

For plasmid transfection, cells were transfected with the indicated plasmids using FuGENE-HD (Promega Biosciences) according to the manufacturer’s instructions. The transfection was carried out at 40% cell density. For 12-well plates in transfection experiments, 1 μg of pDNA and 2 μl of FuGENE-HD were used.

For RNA interference, FlexiTube siRNA targeting NRF2 (SI03246614 [#1, Target sequence is 3’ UTR region.], SI03246950 [#2] and SI4223009 [#3]) (QIAGEN) was transfected into cells at 20 nM using Lipofectamine RNAiMAX (Invitrogen). A control siRNA (ALLStars negative control siRNA, 1027281, QIAGEN) with no known mammalian homology was used as a negative control. The transfection was also performed at 40% cell density. For 12-well plates in transfection experiments, 2 μl of siRNA (final concentration of 20 nM) and 4 μl of Lipofectamine RNAiMAX were used.

Luciferase activity of transfected cells was measured using a luciferase reporter assay system (Perkin Elmer) and Fluoroskan Ascent FL (Thermo Scientific, Lafayette, CO, USA). The luciferase activity was normalized to co-transfected GFP fluorescence.

### Real-time PCR

Total RNA was prepared using an SV Total RNA Isolation System (Promega Biosciences). First-strand cDNA synthesis was performed with total RNA using a SuperScript III First-Strand Synthesis System for RT-PCR (Invitrogen). Synthesized cDNA was used for PCR analysis using TaqMan Fast Uviversal PCR Master Mix (Applied Biosystems) with TaqMan Gene Expression Assay targeting PINK1 (Hs00260868_m1), NRF2 (NFE2L2) (Hs00975961_g1) and glyceraldehyde 3-phosphate dehydrogenase (GAPDH) (Hs02758991_g1) (Applied Biosystems). Relative expression levels were analyzed using StepOnePlus (Applied Biosystems) and they were calculated using the ΔCt method and normalized against GAPDH as an internal control.

### Western blot analysis

Western blot analysis was performed under conventional conditions after lysing cells using SDS sample buffer with PhosphoSTOP (Roche Applied Science, Indianapolis, IN, USA). Ten μg of protein extracts was separated by SDS-polyacrylamide gel electrophoresis and electro-transferred onto an Immobilon membrane (Millipore). To detect the immunoreactive proteins, we used HRP-conjugated anti-mouse or anti-rabbit secondary antibodies (Cell Signaling Technologies) and Pierce Western Blotting Substrate Plus (Thermo Scientific).

### Electrophoretic mobility shift assay (EMSA)

Nuclear extracts from SH-SY5Y cells were prepared using Nuclear and Cytoplasmic Extraction kit (WaKo). With these protein samples, gel shift analysis was carried out using the LightShift Chemiluminescent EMSA kit (Thermo Fisher Scientific, Waltham, MA). The four types of double-stranded ARE probes, candidates for NRF2 binding, which were located from distal through proximal human PINK1 promoter were used (ARE1: 5’-TGTGCTGACTCAGCACACG-3’ (-2637 to -2619 bp), ARE2: 5’-TTCCATGAGTGAGCCTGTG-3’ (-1747 to -1729 bp), ARE3: 5’-TGTGGTGAGCAGGCCTATG-3’ (-665 to -647 bp), ARE4: 5’-CTGCCTGAACCGGCAAGCC-3’ (-240 to -222 bp)). The nuclear extracts were mixed with each 5’-biotin labeled probe and the reaction mixtures were incubated on ice for 10 min. For supershift analysis, rabbit anti-NRF2 antibody (12721, Cell Signaling Technologies) or control rabbit IgG (R&D Systems, Minneapolis, MN)) was further added to the reaction mixture. DNA–protein complexes were then separated by electrophoresis in a 5% polyacrylamide gel under non-denaturing conditions, blotted onto a positive charged Nylon membrane (GE Healthcare), and subjected to cross-linking with UltraViolet (UV) light.

### Chromatin immunoprecipitation (ChIP) assay

ChIP was performed with cross-linked chromatin from 5 x 10^6^ cells, and 5 μl of rabbit anti-NRF2 antibody (12721, Cell Signaling Technologies) using SimpleChIP enzymatic chromatin IP kit (Cell Signaling Technologies). The enriched DNA was measured by PCR using ARE1 primers (Fw: TAAGATAAGGGGGGTTGCAGAAG, Re: CTCAAGGATGGTGGTTTTTATGG, 159 bp), ARE2 primers (Fw: TCTTGAACTCCTGGCCTCAAGCGAT, Re: ACATTCCAGTAACCACAGGCTCAC, 162 bp), ARE3 primers (Fw: GTTTCAGTAATGTGCGTGTCGTGC, Re: AAGACCCCAAGACAAATCTCACCC, 157 bp) or ARE4 primers (Fw: AAGACGTAAAGGGTCTGGCACCAT, Re: CTCTAGCAGTGACTTTCCCTTTGC, 161 bp).

### Cell viability assay and ROS assay

The CellTiter 96 aqueous one solution cell proliferation assay (Promega Biosciences) was used for cell viability assessments. According to the manufacturer’s instructions, cells were incubated with CellTiter 96 aqueous one solution reagent for 3 h. The absorbance of 450 nm was analyzed using an iMark microplate absorbance reader (Bio-Rad). ROS-Glo H_2_O_2_ assay (Promega Biosciences) was used for an ROS assay. The cells were incubated with test compounds and H_2_O_2_ substrate solution for 6 h and then ROS-Glo detection solution was added. The luminescence was observed using Fluoroskan Ascent FL.

### Statistical analysis

Prior to statistical analysis, each experiment was repeated three times. The results are expressed as means +/- S.D. For comparison, analysis of variance (ANOVA) was used. If the ANOVA showed a significant difference, the Bonferroni procedure was used as a post hoc test. *p* values of less than 0.05 were considered statistically significant.

## Results

### Transcriptional up-regulation of *PINK1* under oxidative stress conditions

**Fig 1:**
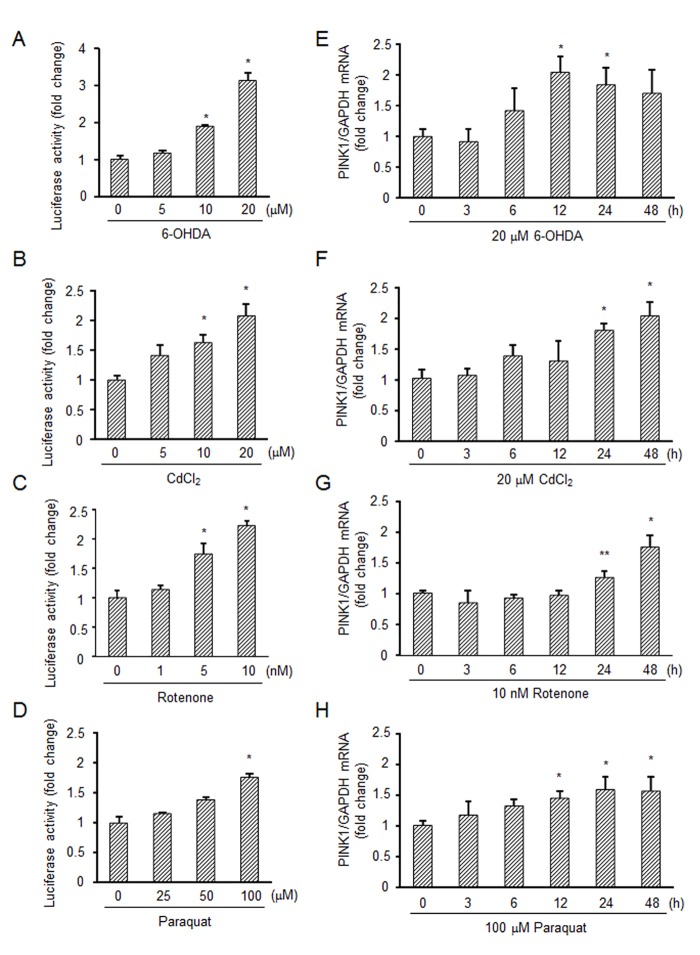
Induction of *PINK1* expression by oxidative stress inducers. (A-D) Luciferase assay using the PINK1 promoter. SH-SY5Y cells were transfected with the PINK1 promoter-luciferase reporter construct (PINK1 pro-luc) and GFP in parallel for 24 h followed by treatment with 0–20 μM 6-OHDA for 48 h (A), 0–20 μM CdCl_2_ for 48 h (B), 0–10 nM rotenone for 48 h (C) or 0–100 μM paraquat for 48 h (D). The luciferase activity was normalized to the fluorescence of GFP in each sample. (E-H) Real-time PCR analysis of *PINK1* mRNA expression. SH-SY5Y cells were exposed to 20 μM 6-OHDA (E), 20 μM CdCl_2_ (F), 10 nM rotenone (G) or 100 μM paraquat (H) at different times. *PINK1* mRNA levels were measured by real-time PCR. Relative expression was determined using *GAPDH* as a housekeeping gene. *, significantly different from the non-treated cells (*p* < 0.01); **, *p* < 0.05.

To examine the effect of oxidative stress on expression of *PINK1*, a reporter assay was carried out using the PINK1 promoter (3 kbp)-luciferase-reporter construct (PINK1 pro-luc). We used human neuroblastoma SH-SY5Y cells for this assay because the cells are often used as *in vitro* models to analyze neuronal function and Parkinson’s disease. SH-SY5Y cells were transfected with PINK1 pro-luc for 24 h and then treated with a neurotoxic reagent, 6-hydroxydopamine (6-OHDA); a metal-stress inducer, CdCl_2_; an inhibitor of mitochondrial respiratory complex I, rotenone; and an ROS inducer, paraquat for 48 h. As shown in Fig 1A–1D, these reagents increased the luciferase activity of PINK1 pro-luc in a dose-dependent manner. We next examined effects of the reagents on expression of endogenous *PINK1* mRNA at regular time intervals. These reagents increased *PINK1* mRNA levels at 12–48 h (Fig 1E–1H). These results indicate that transcriptional expression of *PINK1* is positively regulated under oxidative stress conditions.

### NRF2-mediated induction of *PINK1* mRNA

**Fig 2:**
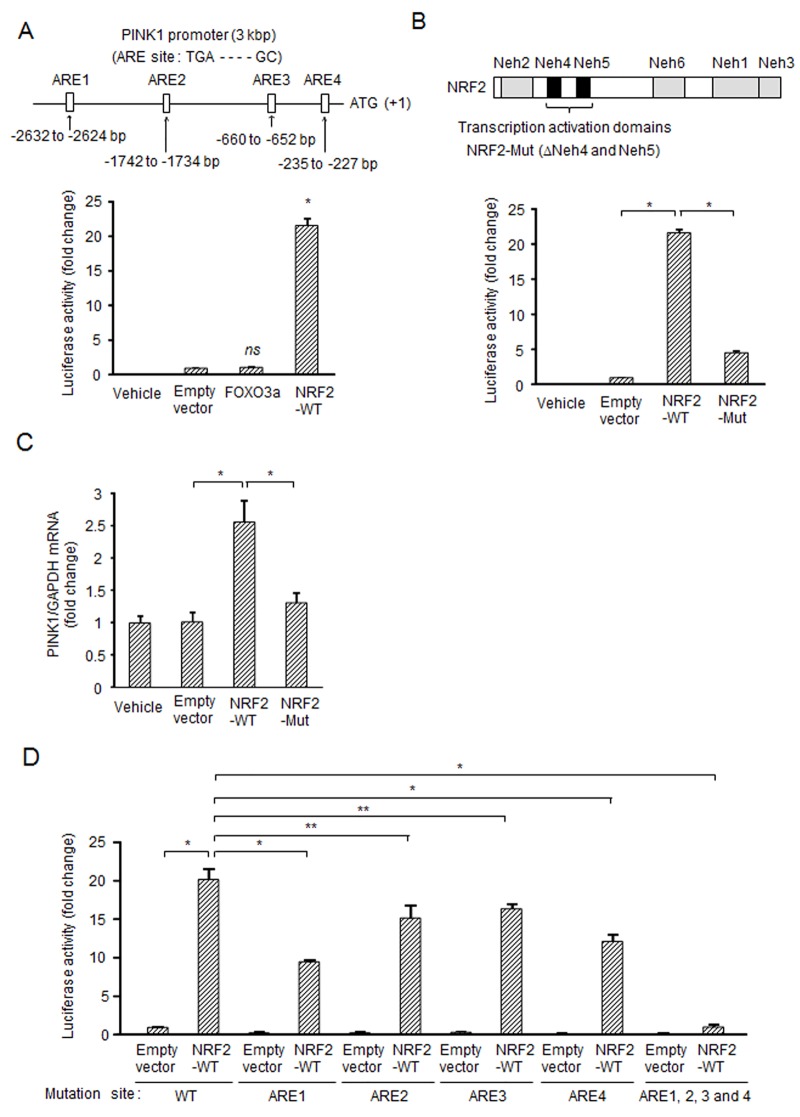
NRF2 regulates *PINK1* expression. (A) Induction of *PINK1* expression by overexpression of NRF2 but not by that of FOXO3a in SH-SY5Y cells. SH-SY5Y cells were transfected with designated constructs and GFP along with PINK1-pro luc (3 kbp) for 48 h. The PINK1 promoter of 3 kbp contains the four NRF2 binding sites (ARE1~4). The luciferase activity was normalized to the fluorescence of GFP in each sample. (B) Requirement of transcription activation domains of NRF2 for induction of *PINK1* expression. Upper schematic diagram shows domains of NRF2. The human *NRF2* gene encodes 605 amino acids and NRF2 has six domains, Neh1 to Neh6. Neh1, DNA binding domain; Neh2, Degron and interaction domain of Keap1; Neh3-5, Transcription activation domains; Neh6, Degron. SH-SY5Y cells were transfected with NRF2-WT or NRF2-Mut lacking Neh4 and Neh5 along with GFP and PINK1 pro-luc for 48 h. The experiment was performed under conditions similar to those described in (A). (C) Up-regulation of *PINK1* mRNA expression by forced expression of NRF2-WT but not by that of NRF2-Mut. *PINK1* mRNA levels were measured by real-time PCR. (D) Requirement of NRF2 binding to the ARE site for induction of *PINK1* expression. SH-SY5Y cells were transfected with designated constructs and GFP along with the pGL4.14-PINK1 promoter (WT, ARE1 mutation, ARE2 mutation, ARE3 mutation, ARE4 mutation or all mutation of ARE1~4) for 48 h. The experiment was performed under conditions similar to those described in (A). *, *p* < 0.01; **, *p* < 0.05; *ns*, not significant.

We next attempted to identify a possible target transcription factor(s) that up-regulates *PINK1* expression. FOXO3a has been reported to control *PINK1* expression [11]; however, forced expression of FOXO3a did not show any increase in luciferase activity of the delivered PINK1 pro-luc in this experimental setting (Fig 2A). This prompted us to further search for a functional sequence(s) of the PINK1 promoter. Sequence analysis showed the existence of putative four antioxidant responsive elements (ARE) in the PINK1 promoter. These ARE candidates (ARE1, -2632 to -2624 bp; ARE2, -1742 to -1734 bp; ARE3, -660 to -652 bp; ARE4, -235 to -227 bp) were matched to the general consensus sequence (TGA-—-—GC), which is the binding site of NRF2. PINK1 pro-luc was used to determine whether the expression of PINK1 is dependent on NRF2. As a result, we found that aberrant overexpression of wild-type NRF2 (NRF2-WT) greatly increased the luciferase activity (Fig 2A). The promoter activity induced by NRF2 was approximately 20-fold higher than that induced by FOXO3a. NRF2-mediated increase in activation of the PINK1 promoter was also observed in different cell lines (S1 Fig). In different cell lines such as HCT-15 and HCT116, FOXO3a also increased the luciferase activity. Based on the homology of cross-species orthologues, NRF2 has been divided into six domains, Neh1 to Neh6 [24], and Neh4 and Neh5 are transcription activation domains [27]. When a deletion mutant of NRF2 lacking the Neh4 and Neh5 domains (NRF2-Mut) was used to assess NRF2 function as a transcriptional regulator for the PINK1 promoter, NRF2-Mut showed only slight activity compared to that of the WT counterpart (Fig 2B). We also confirmed that *PINK1* mRNA level was increased by overexpression of NRF2-WT but not by overexpression of NRF2-Mut (Fig 2C). To determine whether lack of ARE sites for NRF2 binding decreases the promoter activity, the PINK1 promoter with mutation of the ARE sites (ARE1~4) were newly constructed. As shown in Fig 2D, promoter activity of all mutant constructs was decreased compared with WT-promoter even when NRF2-WT was overexpressed. Mutation of ARE1 reduced the promoter activity with the highest level. By mutating all ARE sites (ARE1~4), the promoter activity was completely diminished (Fig 2D). To examine physical binding of NRF2 to the ARE sites, electrophoretic mobility shift assay (EMSA) was employed. With the DNA probes matched to the ARE sites (ARE1~4) in the PINK1 promoter, we found that all ARE probes was able to form complex with proteins in nuclear extracts of SH-SY5Y cells (S2A Fig). The shifted bands were of the NRF2, because supershifted bands were detected by the NRF2 antibody. We also tried to detect intranuclear binding of NRF2 to the ARE sites in the PINK1 promoter. By ChIP analysis, we found that overexpression of NRF2-WT increased the binding of NRF2 to the ARE sites in cells (S2B Fig). These results indicate that NRF2 transcriptionally up-regulates *PINK1* expression via binding to the ARE sites of the PINK1 promoter.

### Involvement of ROS in the NRF2-PINK1 signaling axis

**Fig 3:**
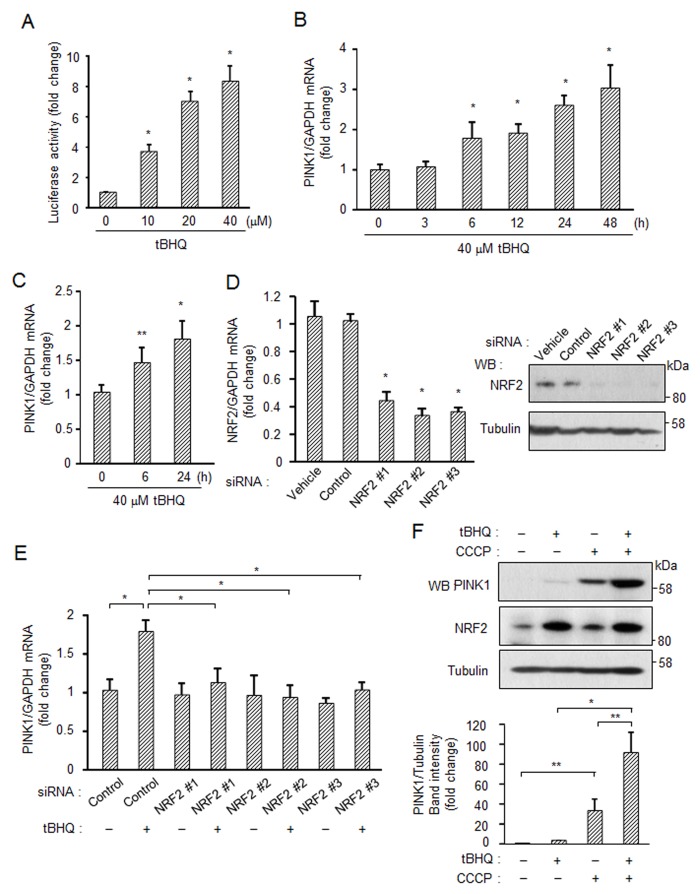
Induction of *PINK1* expression by endogenous NRF2. (A) Activation of endogenous NRF2 and induction of *PINK1* mRNA by an NRF2-activating compound, tBHQ. SH-SY5Y cells were transfected with PINK1 pro-luc and GFP for 24 h followed by treatment with tBHQ for 48 h. (B) SH-SY5Y cells were exposed to 40 μM tBHQ at different times. *PINK1* mRNA levels were measured by real-time PCR. (C) Induction of *PINK1* expression in human matured neurons. Human iPSC-derived neuronal stem cells were cultured in maturation medium for 14 days and were then treated with 40 μM tBHQ for 6 or 24 h. *PINK1* mRNA levels were measured by real-time PCR. (D) Down-regulation of NRF2 using specific siRNAs. (E) Knockdown of NRF2 suppresses *PINK1* expression induced by tBHQ. SH-SY5Y cells were transfected with indicated siRNA for 48 h followed by treatment with 40 μM tBHQ for 6 h. *PINK1* mRNA levels were measured by real-time PCR. (F) tBHQ increases PINK1 protein levels under mitochondrial depolarized condition. SH-SY5Y cells were cultured with 40 μM tBHQ for 24 h and were then treated with 10 μM CCCP for 3 h. Band intensity of PINK1 and tubulin was measured by image J. Prior to statistical analysis, western blotting was repeated three times (Fig 3F and S3C Fig). *, *p* < 0.01; **, *p* < 0.05.

We next used *tert*-Butylhydroquinone (tBHQ), a strong inducer of NRF2 [28], to examine whether endogenous NRF2 regulates *PINK1* expression. As shown in Fig 3A, tBHQ increased the luciferase activity of PINK1 pro-luc in a dose-dependent manner. Real-time PCR analysis showed that tBHQ increased *PINK1* mRNA levels from 6 h to 48 h (Fig 3B). Almost the same results were obtained using stable cell lines expressing PINK1 pro-luc (S3A and S3B Fig). Induction of *PINK1* mRNA by tBHQ was also confirmed in mature neurons derived from human iPS cells (Fig 3C). To evaluate whether the induction of *PINK1* mRNA by tBHQ depends on NRF2 activity, we employed three types of siRNAs against NRF2. The siRNAs used were all confirmed to have suppressive effects on NRF2 expression in both mRNA and protein levels (Fig 3D). Using the validated NRF2 siRNAs, we found that the tBHQ-induced *PINK1* mRNA level was reduced to almost the basal level (Fig 3E). In protein level, PINK1 is constitutively degraded by PARL, a mitochondrial proteinase, under normal conditions, while PINK1 escapes from PARL-processing and accumulates on mitochondria under mitochondrial depolarized conditions [29]. To examine possible linking between mRNA and protein levels of PINK1, SH-SY5Y cells were treated with tBHQ and CCCP, a strong inducer of mitochondrial depolarization (Fig 3F). Single tBHQ-treatment caused slight increase in PINK1 protein. Single CCCP-treatment induced strong accumulation of PINK1 protein by depolarization of mitochondria. tBHQ + CCCP supplied much higher level of PINK1 protein than that supplied by single CCCP-treatment (Fig 3F). These results indicate that NRF2-derived *PINK1* mRNA expression is important to regulate the cellular PINK1 level.

**Fig 4:**
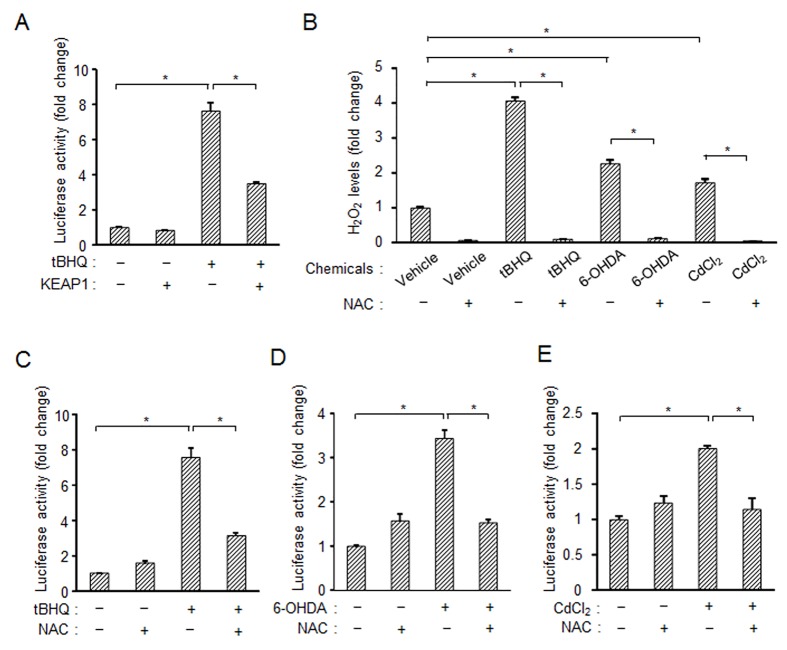
Involvement of ROS in induction of *PINK1* expression by NRF2. (A) Overexpression of KEAP1 suppresses *PINK1* expression induced by tBHQ. SH-SY5Y cells were transfected with N-terminal HA-tagged KEAP1 along with PINK1 pro-luc and GFP for 24 h followed by treatment with 40 μM tBHQ for 48 h. PINK1 promoter activity was measured by a luciferase assay. (B) An ROS scavenger, NAC, inhibits production of H_2_O_2_ induced by chemicals. SH-SY5Y cells were treated with 2 mM NAC along with designated compounds for 6 h. H_2_O_2_ was detected using an ROS-Glo H_2_O_2_ assay. (C-E) NAC inhibits induction of *PINK1* expression induced by 40 μM tBHQ (C), 20 μM 6-OHDA (D) or 20 μM CdCl_2_ (E). These experiments were performed under conditions similar to those described in (A). *, *p* < 0.01.

We also examined other NRF2 inducers for *PINK1* expression. NRF2 inducers including sulforaphane [30], caffeine [31] and curcumin [32] induced increased activity of the PINK1 promoter (S4 Fig). Since these chemical substances including tBHQ are known to inactivate KEAP1, a negative regulator of NRF2, through oxidative stress-mediated ROS production in mitochondria [33], we further investigated an event triggering activation of NRF2 and subsequent *PINK1* induction. Forced expression of aberrant KEAP1 efficiently suppressed the luciferase activity of PINK1 pro-luc induced by tBHQ (Fig 4A). Treatment of the cells with tBHQ increased production of H_2_O_2_, one of the ROS, and the anti-oxidant *N*-acetylcysteine (NAC) quenched the produced H_2_O_2_ (Fig 4B). This NAC treatment effectively suppressed tBHQ-induced PINK1 expression (Fig 4C). Interestingly, in other cases of stimulation with oxidative stress inducers, 6-OHDA and CdCl_2_, the chemical-enhanced production of H_2_O_2_ and PINK1 was greatly suppressed by pretreatment with NAC (Fig 4B, 4D and 4E). These results suggest that ROS production in damaged mitochondria is a critical step for activation of NRF2, which is caused by ROS-mediated inactivation of KEAP1, eventually leading to the transcriptional up-regulation of *PINK1*.

### Cytoprotective function of PINK1 induced by NRF2

**Fig 5:**
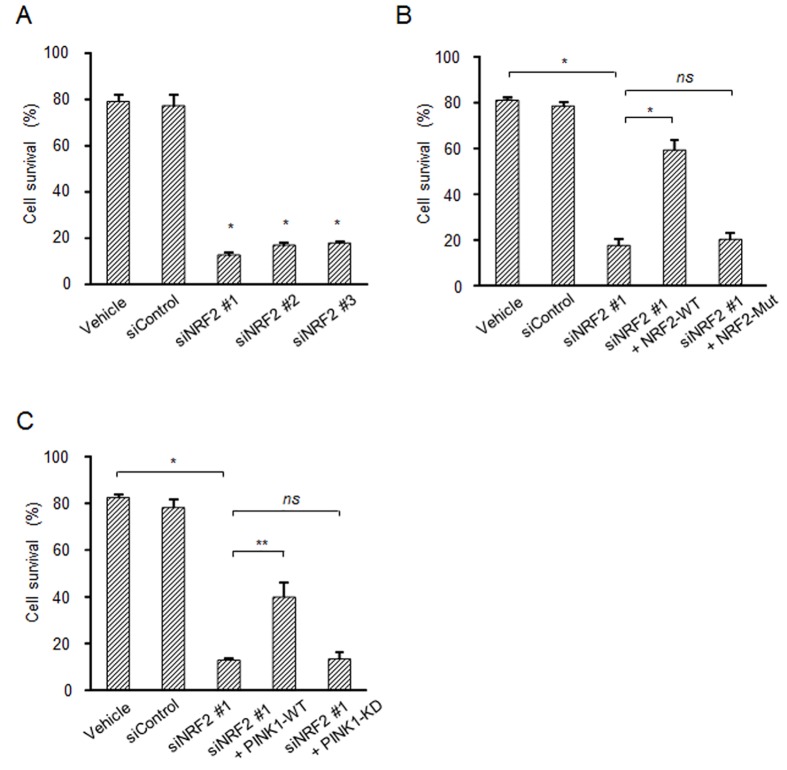
PINK1 induced by NRF2 protects cells against oxidative stress. (A) Down-regulation of NRF2 reduces cell survival. SH-SY5Y cells were transfected with a control or NRF2 siRNA for 48 h followed by treatment with 20 μM 6-OHDA for 24 h. (B and C) Experiments on rescue of NRF2 knockdown by overexpression of NRF2 or PINK1. SH-SY5Y cells were transfected with NRF2-siRNA for 24 h, and then the treated cells were further transfected with indicated NRF2 or PINK1 pDNAs for 24 h followed by treatment with 20 μM 6-OHDA for 24 h. The cell viability was determined by the CellTiter 96 aqueous one solution cell proliferation assay. *, *p* < 0.01; **, *p* < 0.05; *ns*, not significant.

We finally examined the physiological significance(s) of PINK1 induced by NRF2 in cells. To study the cytoprotective function of the NRF2-PINK1 axis against oxidative stress-stimulated cell death, NRF2 was down-regulated using three types of siRNAs in SH-SY5Y cells. Knockdown of NRF2 markedly reduced the ratio of cell survival with 6-OHDA treatment (Fig 5A). Over-expression of NRF2-WT, but not that of NRF2-Mut, rescued the reduction of cell survival caused by 6-OHDA treatment (Fig 5B). We then examined the involvement of PINK1 in this cytoprotective machinery. We used pDNAs expressing the wild type of PINK1 (PINK1-WT) or kinase-dead type of PINK1 (PINK1-KD) for an experiment on rescue of NRF2-knockdown. Over-expression of PINK1-WT, but not that of PINK1-KD, partially rescued the reduction of cell survival under the NRF2-knockdown condition (Fig 5C). These results indicate that PINK1 induced by NRF2 has a role in the protective machinery against oxidative stress-associated cell death.

## Discussion

In this study, we demonstrated that NRF2 functions as a critical transcriptional up-regulator of the *PINK1* gene in response to various stresses. *PINK1* transcriptionally up-regulated by NRF2 has a protective effect on mitochondria, giving cells an advantage for cell survival.

Based on the results of differential gene expression analysis of metastatic cancer cells, we previously showed that the expression levels of *PINK1* were much higher in cancer cell lines with higher metastatic potential than in cancer cell lines with lower metastatic potential [34]. NRF2 expression level also positively correlates with tumor grade. A high expression level of NRF2 stimulates metastatic potential and enhances survival of cancer cells [35, 36]. The present findings regarding the functional link between NRF2 and PINK1, therefore, may also be relevant to cancer progression.

The induction rate of *PINK1* expression by NRF2 differed depending on the cell line (Fig 2A and S1 Fig). It has been reported that NRF2 forms heterodimers with small Maf proteins (MafF, MafG, and MafK), binds to ARE, and activates transcription of target genes [15]. The different expression patterns of small Maf proteins may affect NRF2-derived *PINK1* expression, since we found a very large individual variation in the expression of Maf proteins in different cell lines (data not shown). This possible link warrants further investigations. Mei *et al*. reported that FOXO3a regulates *PINK1* expression in T-lymphocytes upon growth factor/serum deprivation [11]; however, the level of *PINK1* expression induced by FOXO3a in SH-SY5Y cells was much lower than that induced by NRF2. We confirmed up-regulation of *PINK1* expression using tBHQ, an NRF2 inducer, in SH-SY5Y and human matured neurons. These results suggest that *PINK1* transcription is differentially regulated in various cell lineages in which NRF2 has a significant role in *PINK1* transcription, especially in neuronal cells under stress conditions.

NRF2 is an antioxidant transcription factor that positively regulates basal and inducible expression of a large number of cytoprotective genes. When cells are in a non-stressed basal state, NRF2 is sequestered in the cytoplasm by KEAP1 through binding between NRF2 and KEAP1. Under an oxidative stress condition, oxidants and electrophiles oxidize cysteine residues in KEAP1, leading to release of NRF2, allowing it to translocate to the nucleus and induce the expression of its target genes. Indeed, we found that NRF2 functions as a novel transcriptional regulator of the *PINK1* gene via activation of the ARE sequence in the PINK1 promoter. This NRF2-PINK1 signaling axis plays a significant role in cell survival, because induction of oxidative stress-stimulated cell death in NRF2-down-regulated cells was rescued by over-expression of PINK1 (Fig 5C). In addition, PINK1 is involved in the maintenance of mitochondrial homeostasis through various mechanisms such as removal of damaged mitochondria and reduction of ROS. It is hence reasonable to assume that NRF2, an antioxidant transcription factor, actually regulates *PINK1* gene expression. Komatsu *et al*. reported that the selective autophagy substrate p62 activates NRF2 through inactivation of KEAP1 [37]. This mitophagy-related pathway may help us to understand how the NRF2-PINK1 pathway is controlled in mitochondrial homeostasis.

Accumulating evidence indicates that mitochondrial dysfunction plays a central role in the pathogenesis of PD. Mitochondrial dysfunction leads to an increase in ROS production, which is closely linked to neuronal cell death. Therefore, it is important to remove dysfunctional mitochondria and then to synthesize fresh mitochondria for appropriate maintenance of mitochondrial homeostasis. For the renewal of mitochondria, the *peroxisome proliferator-activated receptor gamma coactivator 1-alpha (PGC-1α)* gene is important. PGC-1α is a master regulator of mitochondrial biogenesis [38, 39]. We found that this gene also contains the ARE sequence of the NRF2-binding site in its promoter region. It is hence possible that NRF2 in turn regulates mitochondrial biogenesis through up-regulation of PGC-1α under stress conditions. This pathway may compensate the lack of mitochondria after mitophagy associated with the NRF2-PINK1 signaling axis.

The above-described molecular pathways to activate NRF2 may not function properly in PD, resulting in insufficient prevention of the progression of neurodegeneration. Thus, NRF2 and the NRF2-triggerd signaling axis could be targets for PD therapy. In fact, modulation of NRF2 has been studied as a therapeutic approach in PD. Treatment with Naringenin, a natural flavonoid compound that causes a dramatic increase in the protein level of NRF2, was shown to have a strong protective effect against 6-OHDA-induced nigrostriatal dopaminergic neurodegeneration [40]. Similarly, other chemical compounds that induced high levels of NRF2 were also reported to protect against neuronal damage in a neurotoxin-induced PD model [41, 42]. Thus, NRF2 has become a potential therapeutic target to prevent the progression of neurodegeneration, and the NRF2-PINK1 pathway is also expected to be an emerging target to counteract mitochondrial dysfunction and its consequences in PD.

## Supporting Information

S1 Fig Induction of *PINK1* expression by overexpression of NRF2 or FOXO3a in different cell lines. (A-E) Indicated cells were transfected with designated constructs and GFP along with the pGL4.14-PINK1 promoter for 48 h. The luciferase activity was normalized to the fluorescence of GFP in each sample. *, significantly different from the control group (*p* < 0.01); **, *p* < 0.05; *ns*, not significant.
(TIF)

S2 Fig Physical binding of NRF2 to ARE site. (A) Nuclear extracts of SH-SY5Y cells were incubated with biotin labelled ARE probes (ARE1~4). Complex formation of proteins and ARE probes was confirmed by EMSA. The existence of NRF2 in the complex was detected by adding of NRF2 rabbit mAb. (B) SH-SY5Y cells were transfected with designated constructs for 48 h. ChIP was performed with cross-linked chromatin from 5 x 10^6^ cells, and 5 μl of NRF2 rabbit mAb using SimpleChIP enzymatic chromatin IP kit. The enriched DNA was measured by PCR using primers targeting the region of ARE sequences in the PINK1 promoter.
(TIF)

S3 Fig Induction of *PINK1* expression by tBHQ. (A) SH-SY5Y cells stably expressing PINK1 pro-luc were treated with tBHQ for 48 h. Luciferase activity was measured using a luciferase reporter assay system (B) Time course of tBHQ effect on PINK1 expression in SH-SY5Y cells stably expressing PINK1 pro-luc. (C) tBHQ increases PINK1 protein levels under mitochondrial depolarized condition. SH-SY5Y cells were cultured with 40 μM tBHQ for 24 h and were then treated with 10 μM CCCP for 3 h. *, significantly different from the non-treated cells (*p* < 0.01).
(TIF)

S4 Fig Activation of endogenous NRF2 and induction of PINK1 expression by NRF2-activating compounds. SH-SY5Y cells were transfected with PINK1 pro-luc and GFP for 24 h followed by treatment with 0–5 μM sulforaphane for 48 h (A), 0–200 μM caffeine for 48 h (B) or 0–10 μM curcumin for 48 h (C). The luciferase activity was normalized to the fluorescence of GFP in each sample. *, significantly different from the non-treated cells (*p* < 0.01).
(TIF)

## Abbreviations

ARE: antioxidant responsive element
KEAP1: Kelch-like ECH-associated protein 1
NRF2: Nuclear factor (erythroid-derived 2)-like 2
PD: Parkinson’s disease
PINK1: PTEN-induced putative kinase 1
ROS: reactive oxygen species.
