# Supplementary figures and images for: NRF2 Regulates PINK1 Expression under Oxidative Stress Conditions
Source: PLoS One. 2015 Nov 10;10(11):e0142438. doi: 10.1371/journal.pone.0142438 (PMC4640816; doi:10.1371/journal.pone.0142438)

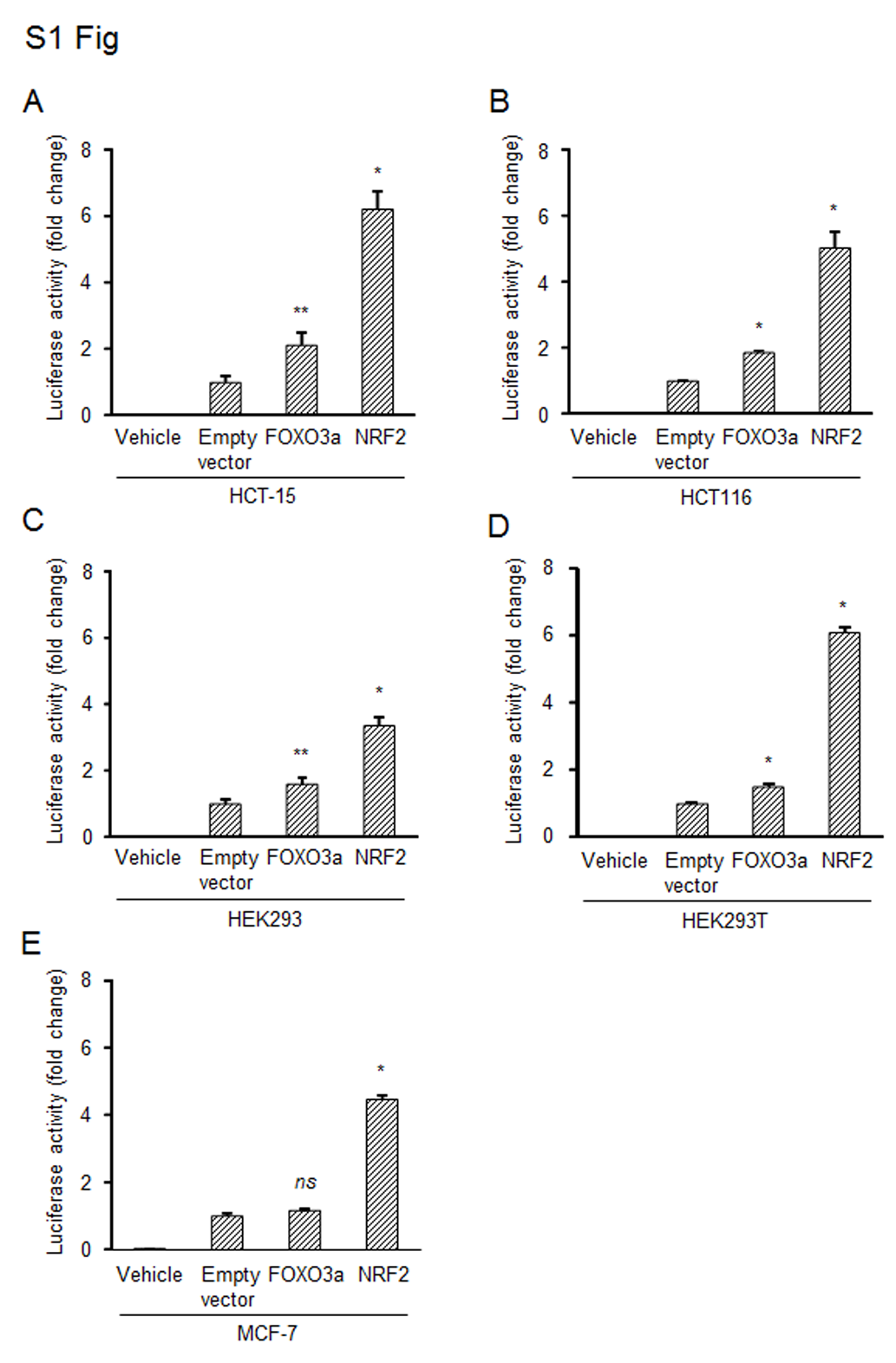

Supplement: S1 Fig — (A-E) Indicated cells were transfected with designated constructs and GFP along with the pGL4.14-PINK1 promoter for 48 h. The luciferase activity was normalized to the fluorescence of GFP in each sample. *, significantly different from the control group (p < 0.01); **, p < 0.05; ns, not significant. (TIF) [file pone.0142438.s001.tif]

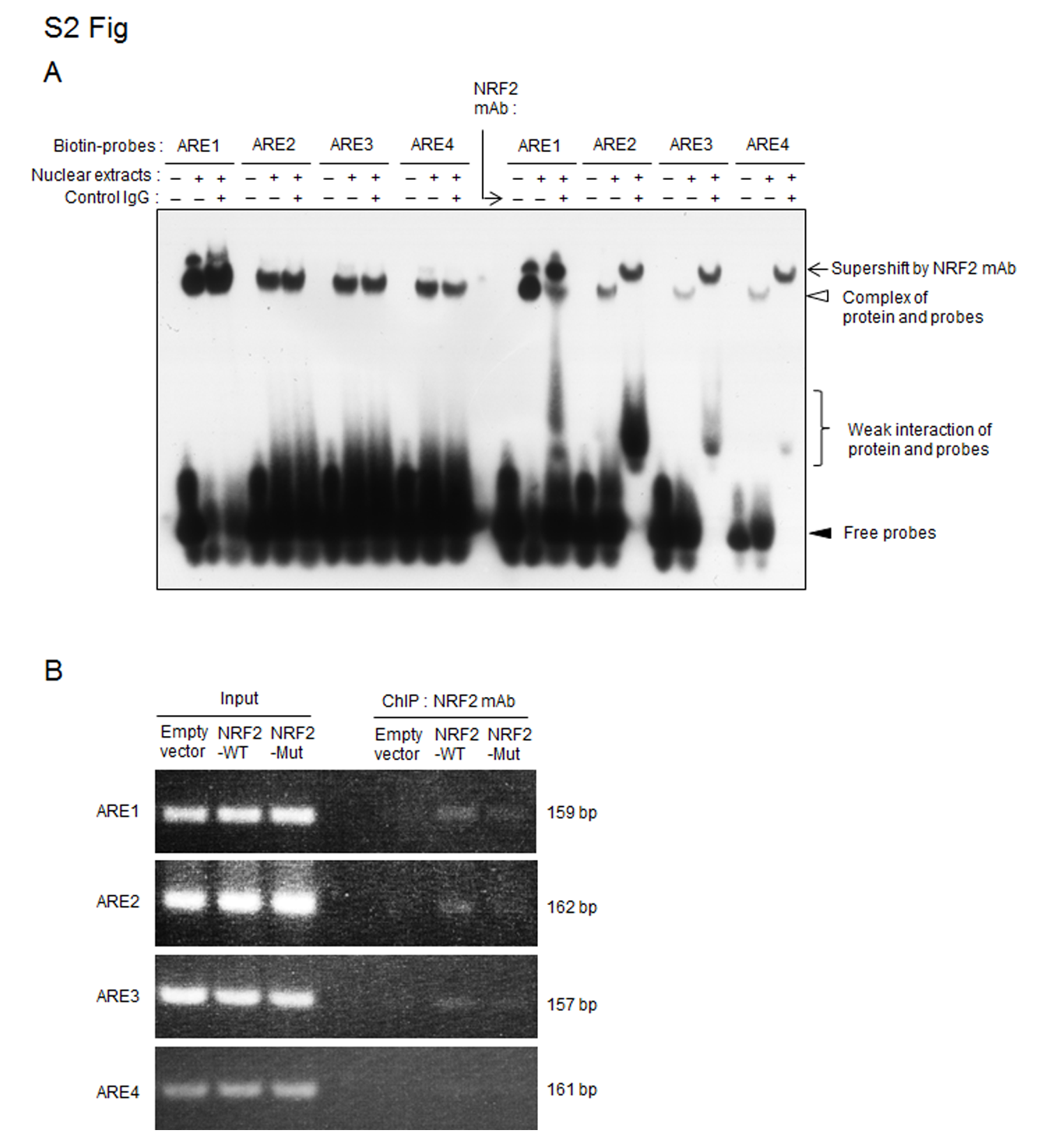

Supplement: S2 Fig — (A) Nuclear extracts of SH-SY5Y cells were incubated with biotin labelled ARE probes (ARE1~4). Complex formation of proteins and ARE probes was confirmed by EMSA. The existence of NRF2 in the complex was detected by adding of NRF2 rabbit mAb. (B) SH-SY5Y cells were transfected with designated constructs for 48 h. ChIP was performed with cross-linked chromatin from 5 x 10^6^ cells, and 5 μl of NRF2 rabbit mAb using SimpleChIP enzymatic chromatin IP kit. The enriched DNA was measured by PCR using primers targeting the region of ARE sequences in the PINK1 promoter. (TIF) [file pone.0142438.s002.tif]

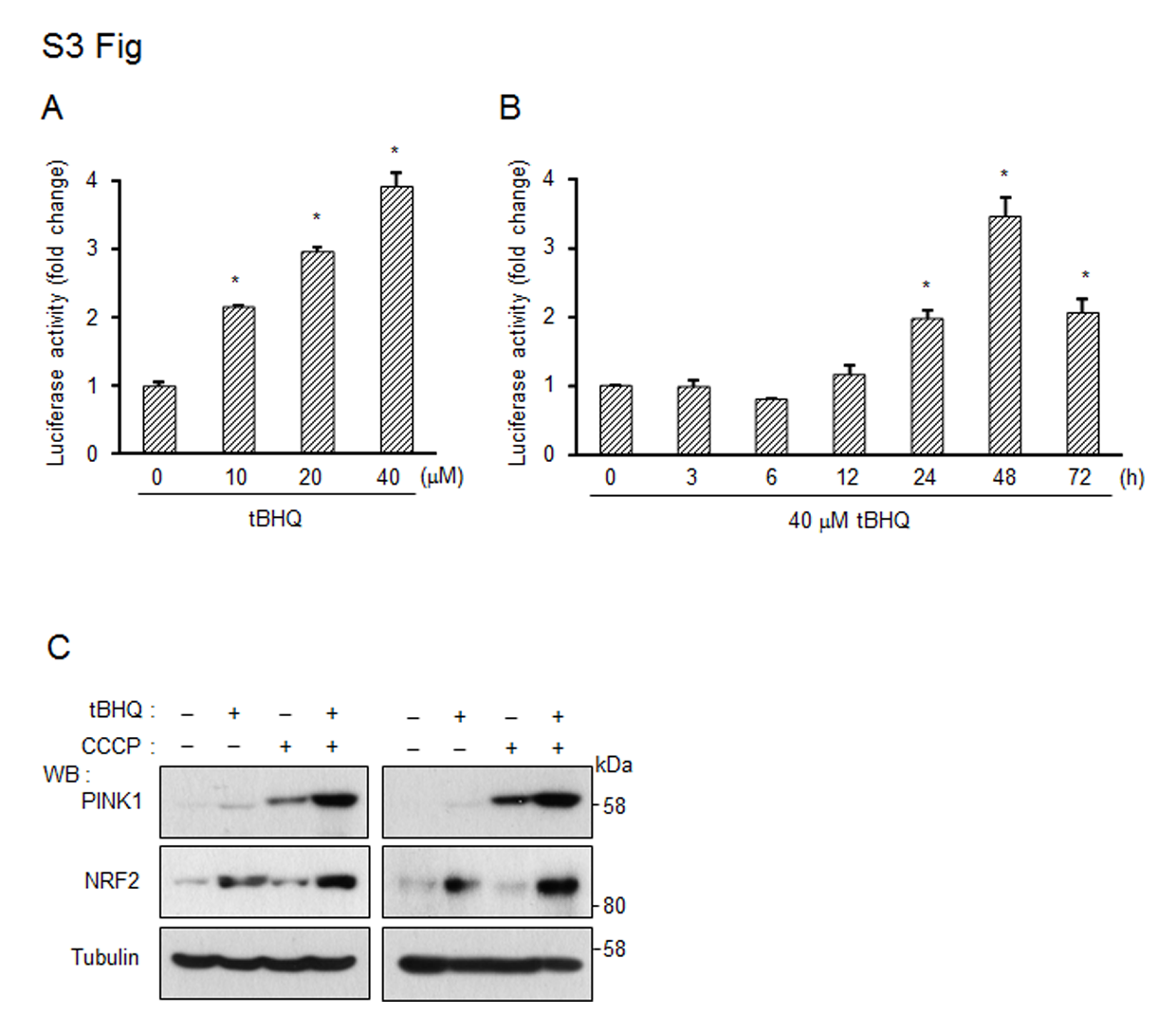

Supplement: S3 Fig — (A) SH-SY5Y cells stably expressing PINK1 pro-luc were treated with tBHQ for 48 h. Luciferase activity was measured using a luciferase reporter assay system (B) Time course of tBHQ effect on PINK1 expression in SH-SY5Y cells stably expressing PINK1 pro-luc. (C) tBHQ increases PINK1 protein levels under mitochondrial depolarized condition. SH-SY5Y cells were cultured with 40 μM tBHQ for 24 h and were then treated with 10 μM CCCP for 3 h. *, significantly different from the non-treated cells (p < 0.01). (TIF) [file pone.0142438.s003.tif]

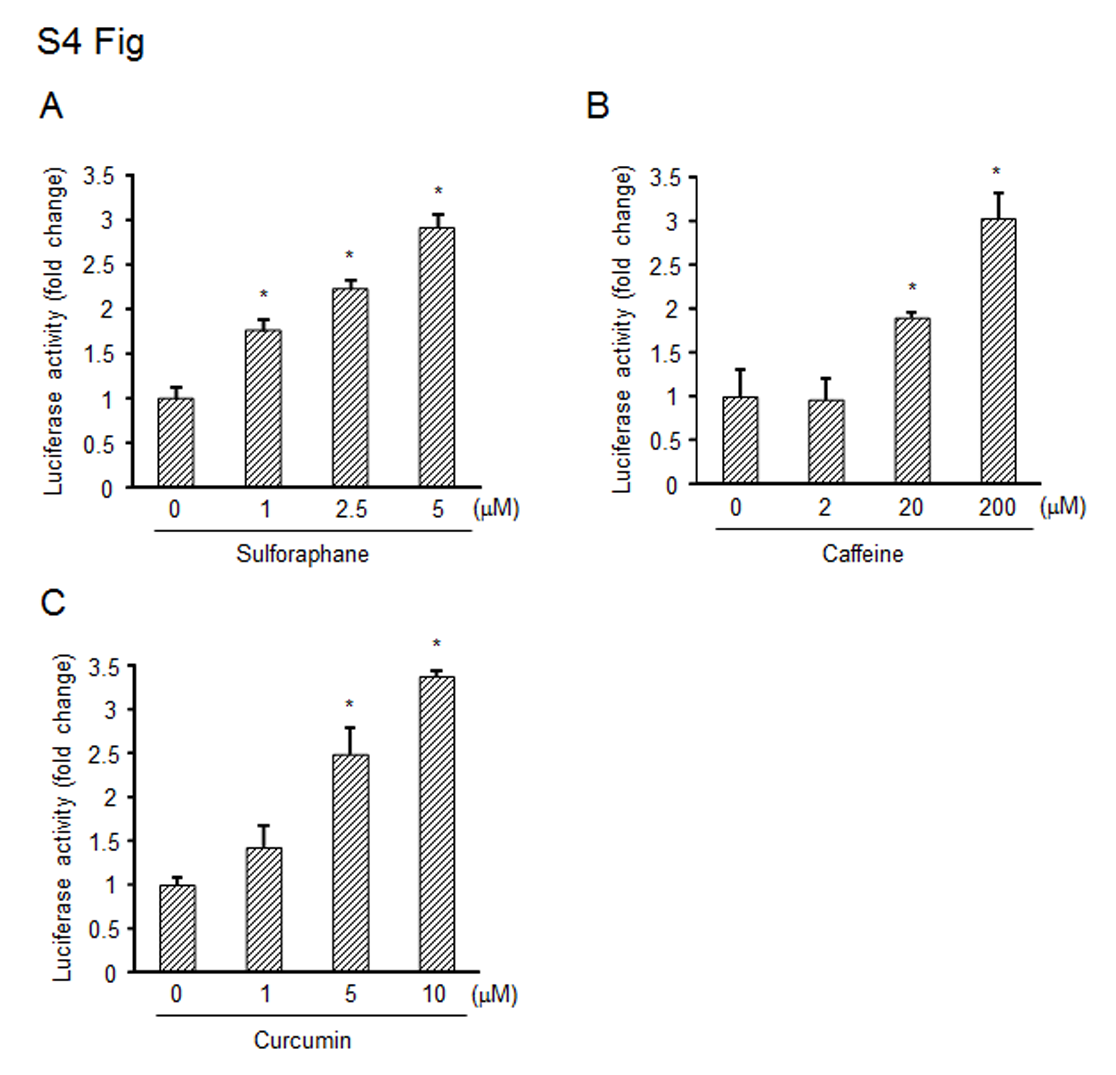

Supplement: S4 Fig — SH-SY5Y cells were transfected with PINK1 pro-luc and GFP for 24 h followed by treatment with 0–5 μM sulforaphane for 48 h (A), 0–200 μM caffeine for 48 h (B) or 0–10 μM curcumin for 48 h (C). The luciferase activity was normalized to the fluorescence of GFP in each sample. *, significantly different from the non-treated cells (p < 0.01). (TIF) [file pone.0142438.s004.tif]
